# A Potent Sybody Selectively Inhibits α-Synuclein Amyloid Formation by Binding to the P1 Region

**DOI:** 10.1021/acs.jmedchem.3c02408

**Published:** 2024-06-06

**Authors:** Dimitra Gialama, Devkee M. Vadukul, Rebecca J. Thrush, Sheena E. Radford, Francesco A. Aprile

**Affiliations:** † Department of Chemistry, Molecular Sciences Research Hub, Imperial College London, London W12 0BZ, U.K.; ‡ Institute of Chemical Biology, Molecular Sciences Research Hub, Imperial College London, London W12 0BZ, U.K.; § Astbury Centre for Structural Molecular Biology, School of Molecular and Cellular Biology, University of Leeds, Leeds LS2 9JT, U.K.

## Abstract

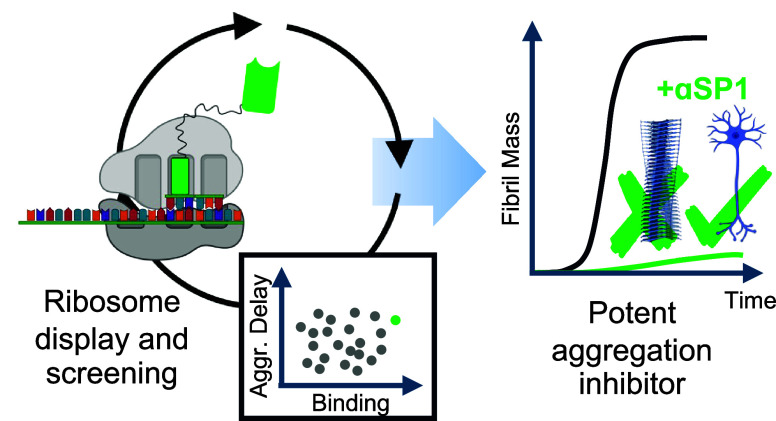

Increasing research efforts focus on exploiting antibodies to inhibit the amyloid formation of neurodegenerative proteins. Nevertheless, it is challenging to discover antibodies that inhibit this process in a specific manner. Using ribosome display, we screened for synthetic single-domain antibodies, i.e., sybodies, of the P1 region of α-synuclein (residues 36–42), a protein that forms amyloid in Parkinson’s disease and multiple-system atrophy. Hits were assessed for direct binding to a P1 peptide and the inhibition of amyloid formation. We discovered a sybody, named αSP1, that inhibits amyloid formation of α-synuclein at substoichiometric concentrations in a specific manner, even within highly crowded heterogeneous mixtures. Fluorescence resonance energy transfer-based binding assays and seeding experiments with and without αSP1 further demonstrate the importance of the P1 region for both primary and secondary nucleation mechanisms of amyloid assembly.

## Introduction

Antibodies are characterized by high binding affinity and specificity for their targets, which makes them ideal probes for biomedical research and appealing as clinical molecules.^1,2^ During the past two decades, antibody-discovery technology has evolved rapidly, and there is now a plethora of *in vitro* selection approaches for generating such molecules, including cell and cell-free display.^3,4^ However, these approaches are generally limited to protein targets that are stable and soluble, with relatively few examples of success with challenging systems, e.g., membrane proteins, intrinsically disordered proteins, and amyloidogenic sequences reported to date.^5−7^

Currently, there is an increasing research effort to develop biologics, including antibodies, that inhibit toxic protein self-assembly, such as the formation of protein fibrils, associated with amyloid deposits involved in neurodegeneration.^8^ One effective strategy for inhibiting amyloid formation is to target the soluble precursors of amyloid fibrils such as monomers or early oligomeric species.^6^ However, non-native states of proteins are commonly heterogeneous and dynamic, creating challenges for antibody generation and issues concerning off-target effects.^9^ It is thus challenging to develop antibodies that can bind amyloid precursors in a preferential manner. Recently, it has been shown that binding to oligomers can be inferred from inhibition studies in which the kinetics of amyloid formation, monitored in the presence of different concentrations of a putative inhibitor, results from highly substoichiometric ratios of an inhibitor to its target sequence.^10,11^

Amyloid formation of the protein α-synuclein (α-syn) is associated with pathologies known as synucleinopathies, which include Parkinson’s disease (PD) and multiple-system atrophy (MSA).^12^ α-Syn is an intrinsically disordered protein that is 140 residues in length and abundantly expressed in dopaminergic neurons.^12^ Under physiological conditions, α-syn regulates the presynaptic terminal size and activity via controlling the distribution of neurotransmitter-containing vesicles.^13^ The sequence of α-syn is composed of three regions, an N-terminal region (residues 1–60), important for the interaction with membranes,^14−16^ the non-amyloid-β component (NAC) region (residues 61–95), which has a high intrinsic amyloid propensity,^17^ and a C-terminal region enriched with acidic residues (residues 96–140), whose truncation promotes amyloid formation.^18^ Recently, a seven-residue motif (P1, residues 36–42) in the N-terminal region has been identified as a “master regulator” of α-syn amyloid formation.^19−22^ Deletion of this motif prevents amyloid formation of α-syn at physiological pH *in vitro*, resulting in the accumulation of prefibrillar species that cannot proceed to amyloid fibrils.^22^ In *Caenorhabditis elegans* (*C. elegans*), deletion of P1 leads to the prevention of age-dependent α-syn aggregation and its associated proteotoxicity.^19^ Additionally, the aggregation of monomeric α-syn into amyloid is inhibited by the engineered β-wrapin AS69, resulting in local folding of the α-syn sequence spanning residues 37–54, a region that includes a large part of the P1 region (residues 36–42), into a β-hairpin conformation.^23^ AS69 is a potent inhibitor of α-syn aggregation into amyloid *in vitro* and in a fruit fly model of α-syn toxicity,^23^ while the AS69-α-syn complex assumes a pivotal role in the inhibition of secondary nucleation as revealed by its potency at substoichiometric concentrations.^24^

Recently, monoclonal antibodies and antibody fragments have been reported to affect amyloid formation.^9,10,25−27^ For Alzheimer’s disease, aducanumab and lecanemab^28−30^ have been fully approved by the Food and Drug Administration (FDA). Donenamab has reached the end of phase III clinical trials,^31^ and their outcome is currently undergoing FDA evaluation. Despite the fact that aducanumab will be discontinued by the manufacturer and there were some questions about its overall effectiveness,^32^ these antibodies represent a significant advancement in Alzheimer’s disease therapeutics as they show efficacy in removing amyloid plaques and in slowing the rate of cognitive decline and memory loss in early stage Alzheimer’s disease patients. Recent studies suggest that lecanemab may be effective in targeting amyloid-β (Aβ) oligomers and potentially mitigating disease progression.^28^ For Parkinson’s disease, prasinezumab, targeting α-syn aggregates, is currently in phase II clinical trials.^27^

One of the limitations of such antibodies is their poor passive adsorption through the blood–brain barrier (BBB).^33^ Furthermore, these antibodies have been associated with secondary effects, e.g., inflammation and bleeding complications within the brain.^34^ Single-domain antibodies (sdAb) are promising therapeutic biomolecules.^35^ Given their small size, they may show better permeability through the BBB. Additionally, these antibody fragments can be further engineered to favor the passage through the BBB by receptor-mediated transcytosis.^36^ As they lack the fragment crystallizable (Fc) domain, sdAbs can be more tolerated by the resident immune system.^37^ Here, we present the development of a synthetic sdAb, i.e., sybody, selected to bind to the α-syn P1 region. We developed an innovative platform for the discovery of P1-specific sybodies. First, we performed ribosome display on a library of sybodies,^7^ to select for those that can bind the P1 sequence using a peptide encompassing the P1 region of α-syn. We then performed a multiparametric screening in which sybody candidates were ranked on the basis of their ability to bind to the P1 peptide over its scrambled counterpart and to inhibit amyloid formation of full-length α-syn. Using seeding assays and experiments involving time-dependent sybody binding, we show that the sybody can inhibit α-syn amyloid formation in a specific and substoichiometric manner by binding to α-syn oligomers formed during the assembly process and to fibrils, preventing secondary nucleation processes. The results highlight the power of sybodies for unpicking the molecular mechanisms of amyloid formation and show that the P1 region of α-syn plays a critical role in multiple steps during amyloid assembly.

## Results and Discussion

### Experimental Strategy

Our goal was to generate a sybody to inhibit α-syn amyloid formation at substoichiometric concentrations in a complex mixture, by specifically interacting with α-syn aggregates. To do so, we targeted the P1 region of α-syn (^36^GVLYVGS^42^) as this sequence has been identified as a “master regulator” of α-syn amyloid formation, with deletion of P1 or mutations of specific residues within its sequence reported to inhibit α-syn amyloid formation and toxicity *in vitro* and in *C. elegans* models.^19,21^

First, we screened a sybody library of concave architecture^7^ against a synthetic peptide with the sequence of P1 by means of three rounds of ribosome display. Then, to isolate the most specific and effective sybody, we combined binding assays on plate and screening of the ability of different sybodies to inhibit α-syn amyloid formation in solution. Specifically, the sybodies were expressed in the *Escherichia coli* (*E. coli*) periplasm and tested directly in the periplasmic extracts for (1) the specificity to bind P1 through an enzyme-linked immunosorbent assay (ELISA) and (2) the inhibition of aggregation of α-syn into amyloid by thioflavin T (ThT) fluorescence assays.

### Ribosome Display and Screening

**Figure 1:**
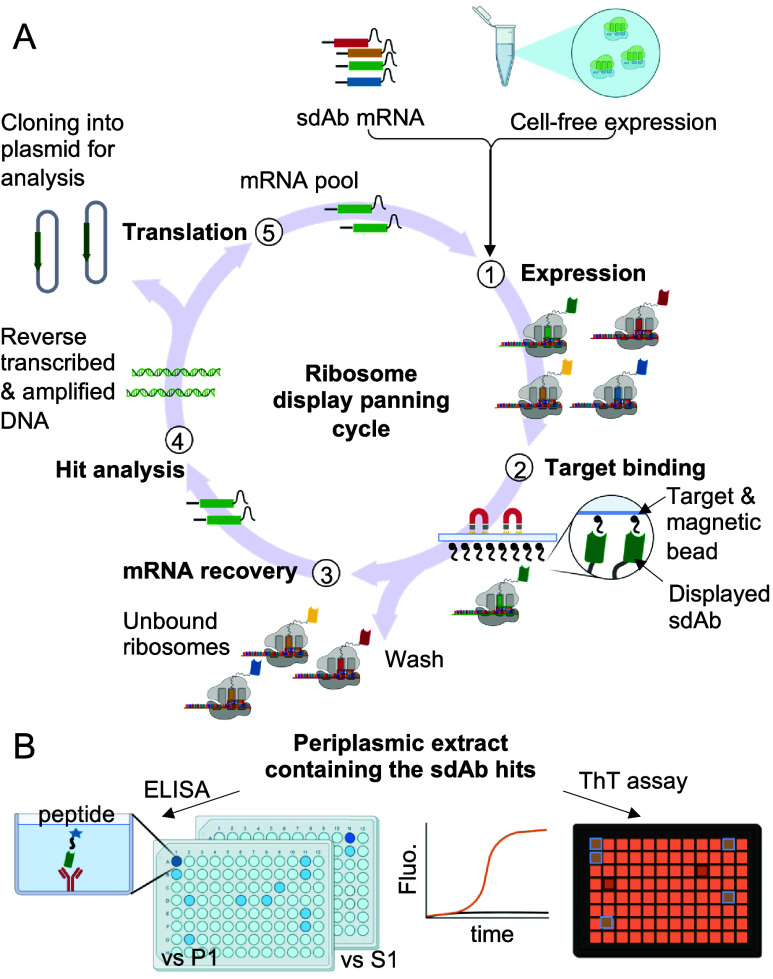
Schematic representation of the ribosome display and multiparametric screening. (A) Three cycles of ribosome display panning against a synthetic peptide encompassing P1 were performed. (B) Sequences from the output of the ribosome display were cloned into a vector in frame with an N-terminal PelB signal sequence that directs the sybodies to the *E. coli* periplasm. Sybodies from 95 single colonies were expressed in the periplasm of *E. coli* and screened. To identify sybodies interacting with P1, periplasmic extracts with sybodies and a control periplasmic extract without a sybody were prepared in a 96-deep-well plate. Then, they were used for binding assays against P1 and its scrambled equivalent peptide S1, and ThT assays were used to assess the inhibition of aggregation of α-syn into amyloid.

A library of sybodies based on an anti-GFP antibody of concave architecture was used.^7,38^ The sybodies in the library contain 15 randomized amino acids, and its theoretical diversity is 8.3 × 10^17^. For the ribosome display experiments, we used purified components from *E. coli* from the commercially available PURE system.^39^ We performed three consecutive rounds of ribosome display against a synthetic peptide with the sequence of P1 from α-syn (Figure 1).

**Figure 2:**
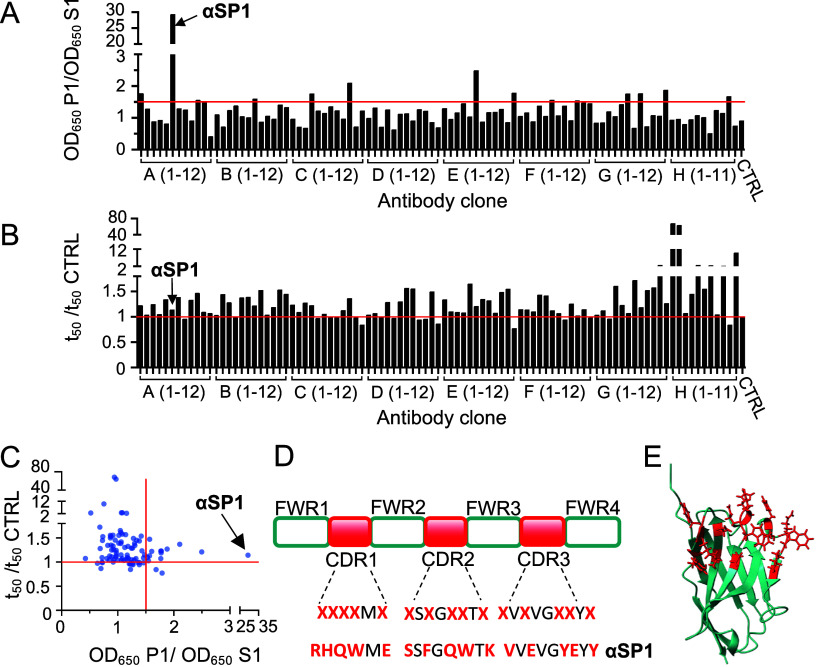
Selection of αSP1 by an ELISA and a ThT assay. (A) Outcome of ELISA screening. Data are represented as the ratio of the optical density (OD) at 650 nm from an ELISA against P1 and from an ELISA against S1. Antibodies with a ratio of >1.5 (red line) were considered potential “hits”. (B) Outcome of the ThT assay. Data are represented as the ratio of the half-time (*t*_50_) of α-syn aggregation in the presence of sybodies and of a control incubation without a sybody (CTRL). The red line indicates no change in *t*_50_ with respect to the control. (C) Plot of the ratios from panels A and B in which αSP1 is highlighted. (D) Representation of the naive library showing the framework regions (FWRs), the complementarity-determining regions (CDRs), and the randomized residues. CDR sequences of αSP1 are also shown. (E) AlphaFold2^41^ prediction of αSP1. The randomized residues are colored red. The PelB signal sequence and tags are not included in this representation.

Ribosome display was subsequently coupled with a binding screen based on a method previously described for sybody discovery against membrane proteins.^7,38^ This method involves fragment exchange (FX) cloning^40^ of genes encoding sybodies into expression vector pSB_init, which generates a fusion of the PelB leader sequence and the sybody domain. Upon overexpression, the sybody is targeted to the *E. coli* periplasm, where disulfide bonds can form. Additionally, the vector appends C-terminal Myc and His tags to the sybody for detection by an ELISA and, subsequently, purification via immobilized metal affinity chromatography (IMAC). To screen for binding, we performed an ELISA against peptide P1 using the periplasmic extracts of *E. coli* clones that each contained a sybody candidate (Figures 1 and 2). To test the specificity for the P1 region, an additional ELISA was performed against the scrambled peptide, S1, containing the P1 residues in a different order (YSGGLVV). The sybodies that had a ratio of the ELISA signal for P1 binding to that of S1 binding of >1.5 were considered as potential “hits”. However, it is crucial that the screening method is based on the property of interest, which is the inhibition of α-syn amyloid formation and not only peptide binding. Hence, an additional step in the screening was to assess the effect of the sybodies on the aggregation of full-length α-syn into amyloid. To achieve this for as many sybodies as possible, recombinant wild-type α-syn (WT α-syn) was incubated in the presence of *E. coli* periplasmic extracts containing the sybodies, hence avoiding the need to first purify the sybodies, and amyloid formation was monitored using ThT fluorescence (Figures 1 and 2 and Figure S1). Given its highly specific binding in which it far outranked other candidates and its significant (but not the largest of all sybodies tested) effect on the *t*_50_ of amyloid formation, αSP1 was taken forward for additional characterization (Figure 2 and Figures S1 and S2).

### Characterization of αSP1

αSP1 was overexpressed in the *E. coli* periplasm and purified using IMAC followed by size exclusion chromatography (Figure S3). Circular dichroism (CD) confirmed the structural integrity of the purified protein (Figure S4), with a resultant spectrum characteristic of immunoglobulin domains.^42^ To verify that αSP1 binds specifically to P1, we assessed its ability to bind to WT α-syn versus a variant of α-syn lacking the P1 region (α-syn ΔP1)^19^ by an ELISA (Figure S5). To do so, we coated the wells of the ELISA plate with the same amount of protein and increasing amounts of αSP1 as a primary antibody in an indirect ELISA setup. We found that the intensity of the ELISA signal decreased when the wells were coated with α-syn ΔP1, supporting the specificity of αSP1 for P1.

**Figure 3:**
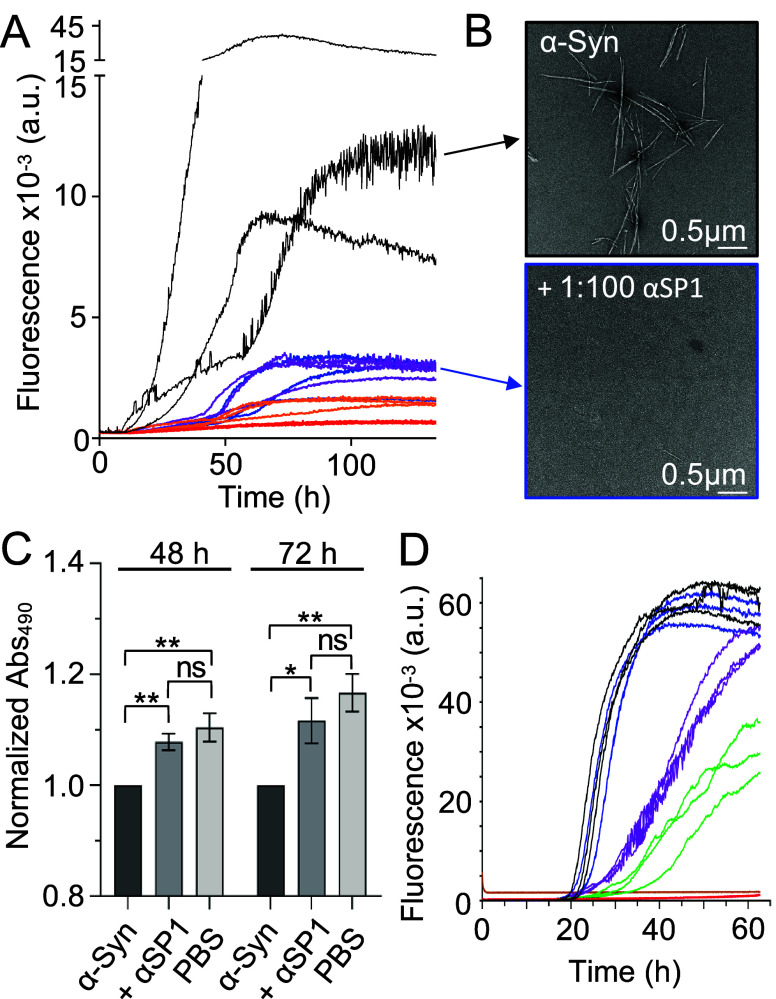
Inhibition of α-syn amyloid aggregation by αSP1. (A) ThT assay of WT α-syn in the presence of different molar ratios of αSP1 (blue for 1:100, purple for 1:50, orange for 1:20, red for 1:10, and black for 0:1, [αSP1]:[α-syn]). Three individual technical replicates are shown. (B) Negative stain TEM images of WT α-syn without (black frame) or with (blue frame) αSP1 (1:100 [αSP1]:[α-syn]). (C) MTS viability assay of SH-SY5Y cells treated with α-syn that has been aggregated in the absence or presence of αSP1 (1:20 [αSP1]:[α-syn] molar ratio) for 48 or 72 h. Data are normalized over the absorbance values of cells treated with α-syn aggregated in the absence of αSP1. The averages of four to seven biological replicates are shown. The error bars represent the standard error of the mean (SEM) of the biological replicates. Each biological replicate is the average of 4–10 technical replicates. Statistical analysis was carried out by one-way ANOVA with Tukey’s multiple-comparison test where *P* > 0.05 (ns), 0.05 ≥ *P* > 0.01 (*), and 0.01 ≥ *P* > 0.001 (**). (D) ThT assay of WT α-syn under secondary nucleation conditions at different molar ratios of αSP1 (red for 1:5, green for 1:10, purple for 1:20, blue for 1:50, and black for 0:1, [αSP1]:[α-syn]). The highest concentration of αSP1 used in the assays (4 μM) was also incubated with preformed fibrils as a control (orange). Three individual technical replicates are shown.

To determine the effect of αSP1 binding on the *in vitro* amyloid formation of α-syn, ThT fluorescence assays were performed on WT α-syn solutions containing increasing substoichiometric concentrations of αSP1. The results showed that amyloid formation of α-syn is almost fully inhibited by αSP1, with a 1:10 αSP1:α-syn molar ratio, increasing the length of the lag phase by ∼2-fold, consistent with the sybody affecting early stages of amyloid formation that include primary nucleation of the assembly reaction (Figure 3A). The effect on the lag phase seems to saturate at a 1:50 αSP1:α-syn molar ratio, but this is likely due to the high sensitivity of primary nucleation to small discrepancies in the initial conditions, e.g., protein concentration and temperature.^43^ As an additional control, the soluble protein fraction at the end point of the experiment was isolated by centrifugation and the amount of soluble α-syn was quantified by Western blotting and densitometry. As shown in Figure S6, the amount of soluble α-syn remaining in solution increases with the concentration of αSP1, in agreement with the ThT results. The inhibitory effect by αSP1 was confirmed by negative stain transmission electron microscopy, which showed fibrillar aggregates at the end of the incubation in the absence of αSP1, and notably fewer fibrils found when α-syn is incubated in the presence of αSP1 at a 1:100 (αSP1:α-syn) molar ratio (Figure 3B).

The experiments described above show that αSP1 is effective at inhibiting amyloid formation of α-syn at low substoichiometric concentrations (even at a 1:100 αSP1:α-syn molar ratio). This observation suggests that inhibition is achieved via preferential interaction of the sybody with α-syn aggregates. α-Syn aggregates are reported to reduce the viability of neurons.^44^ Thus, we assessed whether αSP1 can prevent this mechanism in SH-SY5Y neuroblastoma cells. Twenty micromolar α-syn was aggregated in the absence or presence of a substoichiometric concentration (1 μM) of αSP1. We then collected aggregation data at 48 and 72 h and exposed SH-SY5Y cells to a 1:1 dilution of the time points in cell culture medium. Following incubation for 24 h, cell viability was assessed by the 3-(4,5-dimethylthiazol-2-yl)-5-(3-carboxymethoxyphenyl)-2-(4-sulfophenyl)-2*H*-tetrazolium (MTS) assay (Figure 3C). The absorbance values of the different conditions were normalized over those obtained for cells incubated with only α-syn. The results show that cells incubated with only α-syn are significantly less viable than those incubated with PBS, confirming that α-syn aggregates are toxic to the cells. Cells that were incubated with α-syn aggregated in the presence of αSP1 showed increased viability compared to that of the cells incubated with only α-syn and viability similar to that of the cells incubated with PBS (Figure 3C). To corroborate this result, we quantified the activity of caspase 3/7, as a readout of apoptosis, of cells exposed to time points collected after aggregation for 72 h using the same treatment protocol. We found that αSP1 significantly reduced the activity of caspase 3/7, leading to a partial recovery with respect to the PBS-treated control (Figure S7). Together, these data indicate that αSP1 can inhibit α-syn toxicity in cellular experiments.

Finally, we investigated the effect of αSP1 on one of the key modes of oligomer formation, secondary nucleation.^45^ To do so, ThT fluorescence assays were performed at pH 4.8 and a low molar ratio of preformed fibrils (PFFs) of α-syn to monomers, i.e., 1:1000, conditions under which amyloid formation is dominated by secondary nucleation on the fibril surface.^45^*In vitro*, this pH disrupts electrostatic interactions within the fuzzy coat surrounding α-syn fibrils, exposing their β-sheet core, and favoring interaction of the monomer with the fibril surface.^46^ Furthermore, this pH is biologically relevant as it mimics that of lysosomes, which are important in PD.^47^ Under these conditions, αSP1 showed a clear inhibitory effect on α-syn amyloid formation at substoichiometric concentrations of αSP1 to α-syn as low as 1:20 (Figure 3D), consistent with aSP1 inhibiting secondary nucleation processes. Note that the homogeneity of the seed size of the PFFs was ensured by brief sonication and confirmed by dynamic light scattering (DLS) (Figure S8), and CD indicated that αSP1 does not undergo any significant structural changes at low pH (Figure S9).

**Figure 4:**
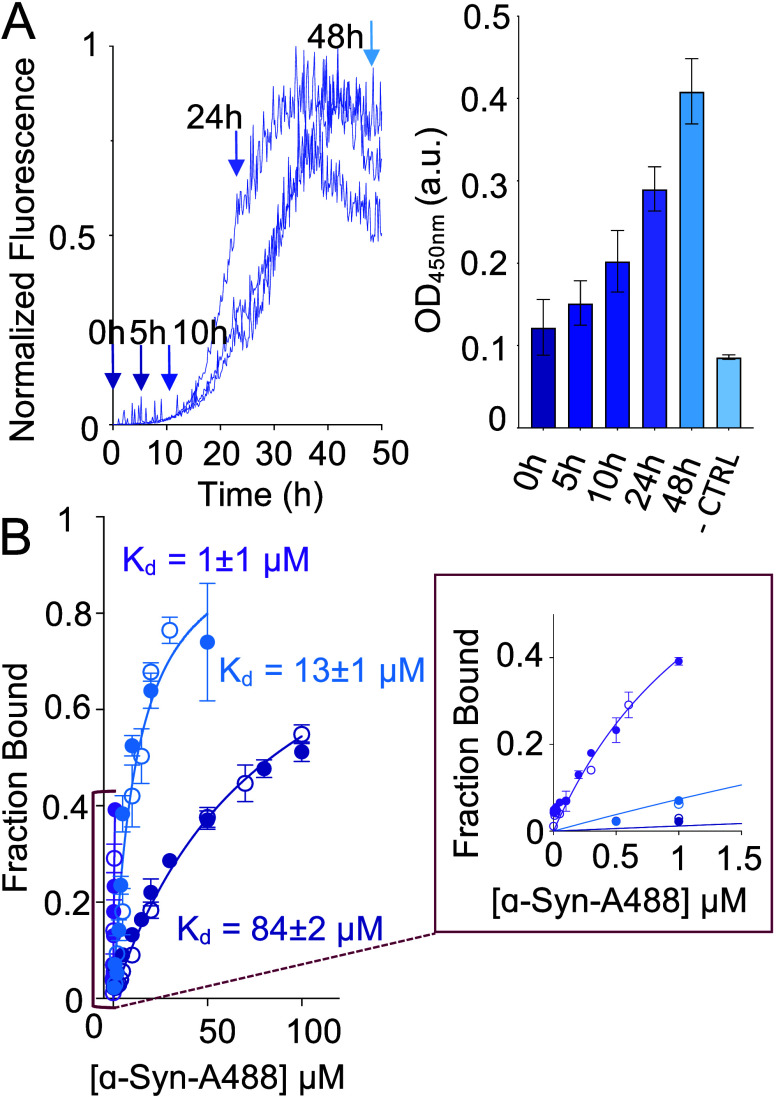
Binding of αSP1 to α-syn aggregates. (A) WT α-syn amyloid formation was monitored by ThT fluorescence (left). Three technical replicates are shown. Samples were collected from the reaction mixture (indicated by the arrows), immobilized on an ELISA plate, and analyzed in an indirect ELISA setup using αSP1 as the primary antibody (right). The level of binding of αSP1 increases with α-syn incubation times, suggesting that this sybody binds oligomeric and fibrillar forms of α-syn. The error bars represent the standard deviation of the mean (SD) of three or four technical replicates. (B) FRET-based titrations to measure the binding of αSP1 to monomeric (blue), oligomeric (purple), and fibrillar (cyan) α-syn. Two biological replicates per condition are shown (denoted with empty or filled circles). Error bars are the standard deviation of three technical replicates. A close-up section of the plot is shown in the red box.

To assess whether αSP1 preferentially binds to α-syn aggregates, we collected samples at different incubation times during the *in vitro* aggregation of WT α-syn. These were then analyzed by an ELISA using αSP1 as the primary antibody (Figure 4A). The results show that αSP1 reactivity is greater for α-syn samples collected after aggregation for 48 h than for samples collected at earlier time points, consistent with the sybody binding preferentially to α-syn oligomers and fibrils.

To further identify which species of α-syn are recognized by αSP1, the binding of αSP1 to monomers and different aggregated species was tested directly using ELISA experiments. For these assays, 80 pmol of αSP1 was immobilized and 160 pmol portions of different α-syn species were assessed for binding. Monomers were collected immediately after size exclusion separation; oligomers were assembled according to established protocols,^48^ and fibrils collected from an in-plate growth assay. The presence of these protein species was assessed by Native PAGE followed by Western blotting (Figure S10). We found that α-syn oligomers showed the highest level of binding to αSP1 compared with monomers and mature fibrils (Figure S11). Next, to derive a *K*_d_ value of binding, we used Förster resonance energy transfer (FRET) titrations (Figure 4B), in which αSP1 was labeled via His tag coordination with NTA-Alexa Fluor 647 as a donor and monomeric, oligomeric, or fibrillar α-syn was labeled with Alexa Fluor 488 as the acceptor (see Materials and Methods). We prepared solutions containing different concentrations of α-syn species and the same concentration of αSP1 and measured the fluorescence emission at 672 nm upon excitation at 493 nm. We determined the apparent *K*_d_ of binding by fitting the resulting data with a one-site specific binding model (see Materials and Methods). We found that αSP1 binds to oligomeric α-syn with the highest affinity (*K*_d_ = 1 ± 1 μM), followed by fibrillar α-syn (*K*_d_ = 13 ± 1 μM), and with a low affinity for α-syn monomers (*K*_d_ = 84 ± 2 μM). Our data support the conclusion that the antiaggregation activity of the sybody is achieved via interaction with aggregated α-syn species, particularly with the oligomers. The lower apparent *K*_d_ for oligomers may be due to avidity, the high degree of solvent exposure of P1 in the oligomers,^22^ or both. Similar affinities have been reported for other anti-amyloid antibodies (IgG), including gantenerumab and aducanamab.^49^ We have previously shown that nanobodies, which are very effective in inhibiting amyloid aggregation, can have high-micromolar-range *K*_d_ values for the monomers and much lower ones for the aggregates.^9,10^ Such binding constants are crucial for them to be selective for aggregated species over the monomers, which is what we have observed for αSP1.

Finally, we tested whether the inhibition of α-syn aggregation by αSP1 at pH 4.8 is associated with a different binding affinity of the sybody for monomeric α-syn. CDR1 of αSP1 contains a His (His35), which is likely protonated at pH 4.8. Similarly, α-syn contains a single His (His50), which is found close to but not in the P1 region. Thus, the binding of αSP1 to monomeric α-syn could be dependent on pH. To test this, we performed FRET titrations to monitor the binding of αSP1 to monomeric α-syn at pH 7.4 and 4.8 (Figure S12). In this case, αSP1 was labeled via Cys coupling using Alexa Fluor 633 maleimide as NTA-Alexa Fluor 647 could dissociate from the His tag at low pH values. We found the *K*_d_ values at pH 7.4 and 4.8 to be similar (96 ± 0.7 μM at pH 7.4 and 80 ± 1.2 μM at pH 4.8), suggesting that the protonation state of His35 (or His50 of α-syn) does not play a key role in the binding of αSP1 to monomeric α-syn. This result supports the conclusion that the αSP1 antiaggregation activity observed at pH 4.8 is due to a preferential binding of the sybody to the aggregated α-syn, which is similar to our observations at pH 7.4.

**Figure 5:**
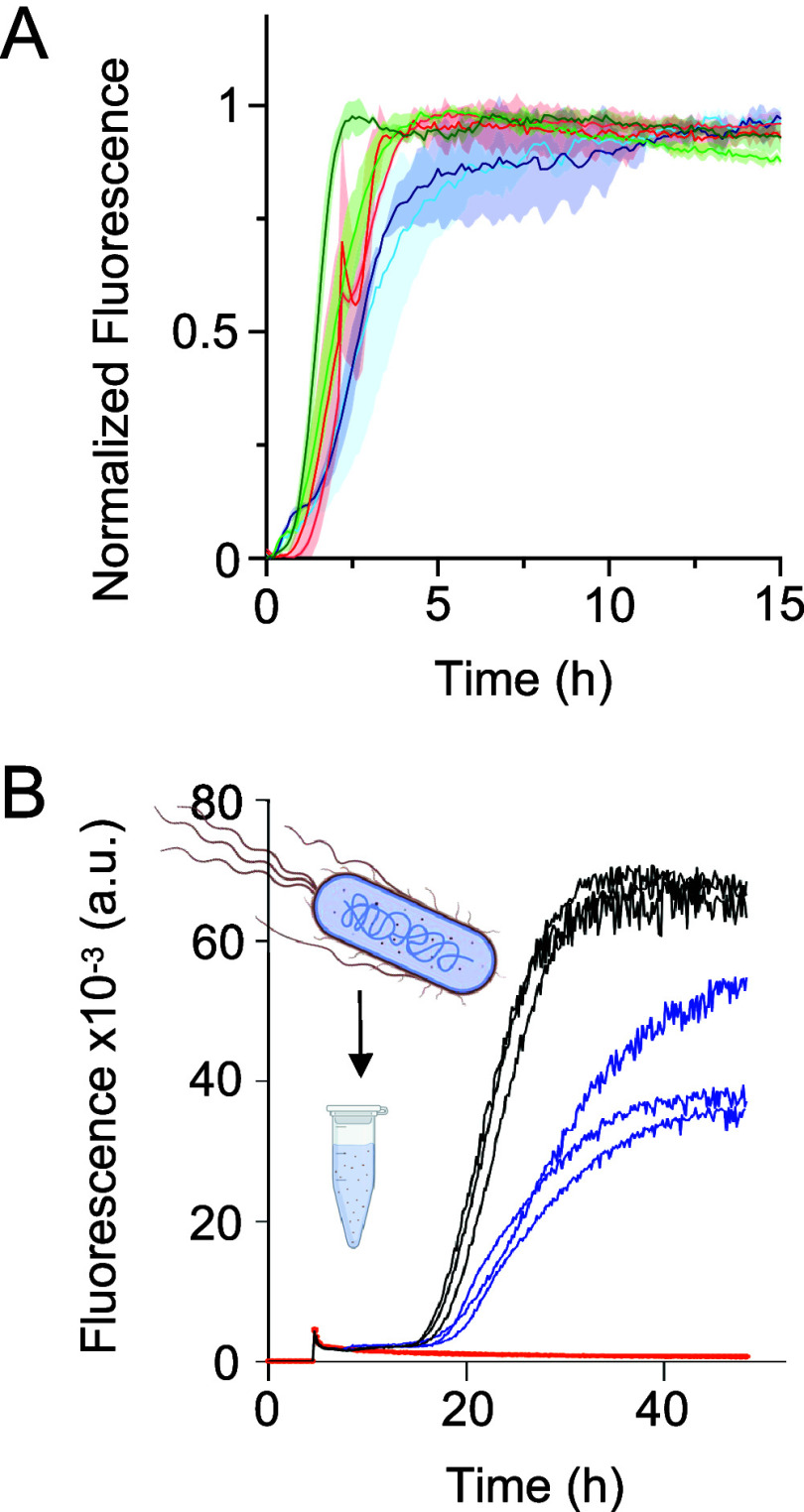
Specificity of αSP1. (A) ThT assay of 1 μM Aβ42 in the absence (dark colors) or presence (light colors) of a 1:10 [αSP1]:[Aβ_42_] molar ratio. The results of three independent experiments are shown. For each independent experiment, the SD of three technical replicates is shown. (B) ThT assay of WT α-syn in cytoplasmic extracts of *E. coli* in the absence (black) or presence (blue) of a 1:100 [αSP1]:[α-syn] molar ratio. The orange trace shows data from a control experiment performed with cytoplasmic extracts of *E. coli* in the absence of α-syn.

Finally, to assess the specificity of αSP1 for α-syn, the ThT assays were performed against another target known to form amyloid, the 42-residue variant of Aβ (Aβ42)^50^ at a 1:10 αSP1:Aβ42 molar ratio (the maximum molar ratio tested for α-syn) (Figure 5A). The results showed no inhibitory effect of αSP1 on the *in vitro* aggregation of Aβ42, confirming that αSP1 inhibits the amyloid formation of α-syn in a specific manner. Finally, we assessed the ability of αSP1 to affect WT α-syn amyloid formation in the presence of an *E. coli* protein extract (Figure 5B) instead of buffer. We found that, even under this condition, αSP1 still inhibits the aggregation of α-syn into amyloid at substoichiometric concentrations, i.e., a 1:100 αSP1:α-syn molar ratio.

## Conclusions

In summary, the results presented show that αSP1 inhibits the self-assembly of α-syn by binding to α-syn oligomers and fibrils, potentially at the surface. Given its small size, αSP1 could have a better permeability through the BBB, although further *in vivo* testing will be required to fully assess the translation potential of the sybody. The results confirm the importance of the P1 sequence as a key controller of α-syn amyloid formation, and via experiments under conditions that favor primary or secondary nucleation processes, we show that this region is required for both nucleation mechanisms of assembly. Interestingly, despite the P1 region being part of the core in most (but not all) α-syn fibril structures determined to date, αSP1 is able to bind to its target.^51^ This highlights the advantages of small single-domain antibodies for accessing and binding their targets and suggests that binding to a β-strand motif might be a common feature of both α-syn oligomers and fibrils. This is in accord with the ability of the P1 region to adopt a β-strand conformation as visualized when bound to an evolved β-wrapin.^23^ Finally, we report a multiparametric screening method for the discovery of antiaggregation sybodies and demonstrate its success in screening for a new anti-amyloid reagent targeted against α-syn.

## Materials and Methods

### Reagents

For cloning purposes, *E. coli* strain XL1-Blue (Agilent) was used. *E. coli* BL21(DE3) from New England Biolabs (NEB) was used for protein overexpression. Synthetic target peptides (P1 and S1) were purchased from GenScript. The peptides were N-terminally biotinylated and C-terminally amidated.

### Plasmid Construction

The DNA primers utilized for the cloning of recombinant DNA are listed in Table 1. All enzymes for cloning of recombinant DNA were purchased from NEB. All plasmids used are listed in Table 2. The pT7-7 α-syn WT plasmid was purchased from Addgene (a gift from Hilal Lashuel, Addgene plasmid 36046).

**Table 1:** Primers Used in This Study

name	sequence (5′ to 3′)	source
RT_Primer	CTTCAGTTGCCGCTTTCTTTCTTG	(38)
Med_FX_for	ATATGCTCTTCTAGTCAGGTTCAGCTGGTTGAGAGCG	(38)
Med_FX_rev	TATAGCTCTTCATGCGCTCACAGTCACTTGGGTACC	(38)
5′_flank_for	CGAAATTAATACGACTCACTATAGGGAGAC	(7)
Medium_ORF_5′_rev	CGCTCTCAACCAGCTGAACCTGACT	(7)
Medium_ORF_3′_for	GGTACCCAAGTGACTGTGAGCGCA	(7)
tolAk_rev	CCGCACACCAGTAAGGTGTGCGGTTTCAGTTGCCGCTTTCTTTCT	(7)
αSA140Cfor	TAAGAAATATCTTTGCTCCCAG (5′-phosphorylated)	this work
αSA140Crev	GCATTCAGGTTCGTAGTCTTGATAC	this work

**Table 2:** Plasmids Used in This Study

plasmid	encodes	source
pRDV5 plasmid containing a loop sybody	loop sybody	sybody generation toolbox (Addgene 1000000160)
pT7-7	WT α-syn	Addgene 36046
pSB_init	N/A	sybody generation toolbox (Addgene 1000000160)
A6pSB_init	sybody αSP1	this work
pET23a backbone containing αSynΔP1	ΔP1 α-syn	(20)
aSA140CpT7-7	A140C α-syn	this work

### Ribosome Display against P1

The first round of ribosome display was performed as previously described in ref (38), and the second and third rounds of ribosome display were performed as described in ref (7) with some modifications. Briefly, for *in vitro* translation, the PUREfrex 2.1 kit and the DS supplement translation mix were used (GeneFrontier Corp.). The kit components were prepared in a total volume of 9.3 μL and incubated at 37 °C for 5 min. The concave RNA library was added to the translation mix (0.7 μL corresponding to 1.6 × 10^12^ mRNA strands) and incubated at 37 °C for 30 min. After formation of the ribosomal complexes, 100 μL of ice-cold WTB buffer [50 mM Tris-acetate (pH 7.5), 150 mM NaCl, and 50 mM magnesium acetate] was supplemented with 40 units of RNase inhibitor (Promega), 0.5% (w/v) BSA, and 5 mg/mL heparin. Three washes with 12 μL of Dynabeads MyOne Streptavidin T1 (Life Technologies) followed with 500 μL of WTB. The Dynabeads were then blocked with WTB-BSA [WTB supplemented with 0.5 % (w/v) BSA] for 1 h followed by three washes with 500 μL of WTB-BSA. To coat the Dynabeads, they were incubated with 100 μL of WTB-BSA containing 500 nM biotinylated P1 for 20 min. After three washes with 500 μL of WTB-BSA, the ribosomal complexes were added to the beads and the mixture was incubated for 20 min. After two washes of the beads with 500 μL of WTB, they were placed in a fresh tube. Another wash with 500 μL of WTB followed, and RNA elution took place via addition of 100 μL of 50 mM Tris-acetate (pH 7.4), supplemented with 150 mM NaCl, 50 mM EDTA (pH 8), and 100 μg/mL yeast RNA and incubation for 10 min at room temperature. The RNeasy micro kit (Qiagen) was used to purify the RNA, which was eluted in 14 μL of RNase-free water. For reverse transcription, the eluted RNA was mixed with 2 μL of RT_Primer at 100 μM and 4 μL of 10 mM dNTPs and heated to 65 °C for 5 min. Then, the sample was cooled on ice, and a 40 μL room-temperature (RT) reaction mixture was prepared according to the manufacturer’s instructions (Affinity Script, Agilent). The reaction mixture was incubated for 1 h at 37 °C and at 95 °C for 5 min. A PCR purification kit (Macherey Nagel) was used to purify the cDNA, which was eluted in 30 μL of elution buffer. PCR amplification followed using 29 μL of the purified cDNA, Q5 High-Fidelity DNA Polymerase (NEB), and primers Med_FX_for and Med_FX_rev for the concave library. Gel purification of the PCR product followed, and the product was used as a template for assembly PCR using megaprimers to append the flanking regions for the *in vitro* transcription step. The megaprimers were prepared as previously described.^7^ To flank the concave and loop sybodies, megaprimers were amplified using the pRDV5 plasmid containing a loop sybody as a template and the 5′_flank_for/Medium_ORF_5′_rev and Medium_ORF_3′_for/tolAk_rev primer pairs.

The DNA fragment of interest was assembled by PCR, as previously described.^7^ Briefly, the sybody pool from RT-PCR (200 ng), the 5′-flank (120 ng), the 3′-flank (360 ng), and primers 5′_flank_for and tolAk_2 (5 mM each) were used in a 100 μL reaction mixture. The assembled PCR product was gel purified and translated into RNA using a 10 μL reaction mixture of the T7 RiboMAX Large Scale RNA Production System (Promega). RNA purification followed using the RNeasy kit (Qiagen), and the purified RNA was used as the input material for the next round of ribosome display. The second round was performed according to the first round using Dynabeads MyOne Streptavidin C1. For the third round, Dynabeads MyOne Streptavidin T1 were used, and after cDNA amplification, the PCR product was subsequently cloned into the pSB_init vector by FX cloning for further analysis as previously described.^38^

### FX Cloning of Hits into Expression Vector pSB_init

First, 300 ng of the ribosome display output and 100 ng of pSB_init were used to clone the sybody hits into expression vector pSB_init. The cloning took place as previously described^38^ except for the ligation and the transformation steps. For ligation, T4 DNA ligase (400 units, NEB) was used at 16 °C for 16 h. For electroporation, BL21(DE3) cells were used.

### Periplasmic Extract and Hit Analysis Using and ELISA

The analysis took place in a 96-well plate as previously described^38^ with a few modifications. Briefly, single sybody clones were expressed in BL21(DE3) cells in 1 mL of terrific broth supplemented with 25 μg/mL chloramphenicol in a 96-deep-well plate with a volume of 2 mL. To prepare periplasmic extracts, the cultures were subsequently centrifuged, and the pellets were resuspended in 100 μL of periplasmic extraction buffer [20% (w/v) sucrose, 50 mM Tris, 25 mM EDTA (pH 8.0), and 0.5 μg/mL lysozyme]. The lysate was diluted with 900 μL of Tris-buffered saline (TBS) supplemented with 1 mM MgCl_2_ and centrifuged to pellet cell debris. The supernatant was used as the periplasmic cell extract for subsequent ELISA and aggregation assay steps.

For the ELISA, MaxiSorp immunoplates (Nunc) were used after overnight coating with 100 μL/well of 5 μg/mL protein A in TBS. After being washed three times with 250 μL of TBS, the plate was blocked with 250 μL of TBS-BSA (TBS supplemented with 0.5% BSA). Every incubation step took place in 100 μL of TBS-BSA for 20 min and involved incubation with a 1:1000 anti-c-Myc antibody dilution (Abcam) followed by the diluted sybody lysate, then 500 nM biotinylated target peptide or biotinylated scrambled peptide, and finally a 1:5000 streptavidin-HRP dilution (Sigma-Aldrich) (Figure 1). After each incubation step, a washing step followed using 250 μL of TBS three times. The ELISA was developed by adding 100 μL/well of 0.1 mg/mL TMB (Tokyo Chemical Industry UK) in 50 mM Na_2_HPO_4_, 25 mM citric acid, and 0.006% H_2_O_2_. The absorbance at 650 nm was measured using a CLARIOstar Plus microplate reader.

### Expression and Purification of Wild-Type, A140C, and ΔP1 α-Syn

WT α-syn DNA contained the TAC136TAT mutation to avoid a Cys misincorporation and subsequent dimerization of the protein.^52^ The plasmid was transformed into *E. coli* BL21(DE3) cells, and WT α-syn expression was induced with 1 mM IPTG at an OD_600_ of 0.7 in lysogeny broth (LB) supplemented with ampicillin (100 μg/mL) at 28 °C overnight. The cells were pelleted and resuspended in buffer A [20 mM Tris-HCl and 1 mM EDTA (pH 8.0)] with a protease inhibitor tablet (EDTA-free, Roche). WT α-syn was then purified as previously described.^53^ Briefly, the crude extract was incubated at 80 °C for 30 min to induce the misfolding and precipitation of all proteins except α-syn. The sample was then centrifuged at 35000*g* for 30 min, and the supernatant was incubated in the presence of 10 mg/mL streptomycin sulfate for 20 min at 4 °C and centrifuged at 35000*g* to precipitate and remove the nucleic acids. The proteins were finally isolated by precipitation using 360 mg/mL ammonium sulfate. Anion exchange chromatography was then carried out using a HiPrep Q HP 16/10 column (Cytiva) and a linear gradient from 0 to 1 M NaCl. Monomeric α-syn was purified by size exclusion chromatography (SEC) in PBS [137 mM NaCl, 2.7 mM KCl, 10 mM Na_2_HPO_4_, and 1.8 mM KH_2_PO_4_ (pH 7.4)] using a HiLoad 16/600 Superdex 75 pg column (GE Healthcare). The protein concentration was determined by measuring the absorbance at 275 nm using a molar extinction coefficient of 5600 M^–1^ cm^–1^.^54^

The same expression and purification protocols were followed for the A140C and ΔP1 α-syn variants. For the purification of A140C α-syn, the final SEC was performed in PBS supplemented with 0.5 mM DTT, which was also used as a storage buffer. The concentration of A140C α-syn was calculated using the same molar extinction coefficient at 275 nm as that of WT α-syn. The concentration of ΔP1 α-syn was determined by measuring the absorbance at 280 nm using a molar extinction coefficient of 4470 M^–1^ cm^–1^ estimated by using ExPASy ProtParam.^54^ The plasmid for A140C α-syn was created by PCR amplification using the Q5 polymerase (New England Biolabs) and the primers listed in Table 1. The template DNA was removed by DpnI digestion for 1 h at 37 °C prior to ligation and transformation in XL1-Blue competent cells (Agilent). The plasmid for ΔP1 α-syn was created in ref (19).

### Generation of α-Syn-Stabilized Oligomers

Solutions enriched with α-Syn oligomers were obtained on the basis of a previously described protocol.^55^ Briefly, 800 μM WT α-syn was lyophilized overnight, resuspended in PBS, and incubated at 37 °C for 20–24 h. The sample was then ultracentrifuged for 90 min at 50 000 rpm (Beckman Coulter Optima MAX-XP Ultracentrifuge with a TLA-55 rotor), and then the supernatant was passed through a 100 kDa cutoff filter four times to remove the monomers. The protein concentration was determined by measuring the absorbance at 275 nm and using a molar extinction coefficient of 7000 M^–1^ cm^–1^.^48^ The formation of oligomers was confirmed by Native PAGE.

### Generation of α-Syn Preformed Fibrils

First, 100–200 μM WT α-syn was incubated in PBS in an Eppendorf tube at 37 °C while being shaken (400–500 rpm) for ∼4 days in the presence 0.02% (w/v) NaN_3_. The fibrils were pelleted by centrifugation (16900*g* for 30 min) and resuspended in PBS. Following repeated centrifugation and resuspension, 0.02% (w/v) NaN_3_ was added, and the fibril concentration estimated by measuring the absorbance at 275 nm (ε_275_ = 5600 cm^–1^ M^–1^) after denaturation in 4 M guanidinium chloride. Following further dilution to 5 μM PFFs in either PBS or 20 mM sodium acetate and 150 mM NaCl (pH 4.8), the fibrils were sonicated at a 20% power, a 5 s pulse, and a 5 s rest for three cycles using a probe sonicator on ice.^56^ The size distribution of the fibrils was measured by DLS using a Zetasizer Ultra instrument (Malvern Panalytical).

### Expression and Purification of αSP1

Sybody αSP1 was expressed in BL21(DE3) *E. coli* cells, which were grown in LB containing 25 mg/mL chloramphenicol to an OD_600_ of 0.7 at 37 °C. The gene encoding the sybody in pSB_init was under the control of the araBAD promoter, and protein expression was induced with 0.02% (w/v) l-arabinose at 22 °C for 18 h. Cells were lysed in PBS supplemented with 30 mM imidazole and one tablet of protease inhibitors per liter of culture using a probe sonicator. Centrifugation at 10000*g* for 15 min was then used to remove cell debris, and the proteins were purified using Ni^2+^ affinity chromatography. Briefly, the supernatant was incubated with a 1.5 mL bed volume Ni-NTA slurry (Qiagen) at room temperature while being constantly agitated. The beads were washed twice with PBS containing 50 mM imidazole at pH 7.5, and the His-tagged antibody was eluted twice with PBS containing 300 mM imidazole at pH 7.5. The Ni-NTA-purified sybodies were dialyzed against PBS overnight at 4 °C to remove imidazole. The sybodies were concentrated using centrifugal filters with a 3 kDa cutoff (Amicon Ultra-4) and separated by SEC using a Superdex 75 Increase pg column (GE Healthcare). The protein concentration was determined by measuring the absorbance at 280 nm using a molar extinction coefficient of 33 920 M^–1^ cm^–1^, calculated using ExPASy ProtParam.^54^ The protein purity (>95%) was assessed by SDS–PAGE (Figure S3).

### Expression and Purification of Aβ42

Aβ42 was purified as previously described.^57^ Briefly, the Aβ42 peptide conjugated to a spider silk domain (called the fusion protein) for solubility purposes was expressed in BL21(DE3) *E. coli* cells. Cultures were grown in Lennox broth supplemented with 50 μg/mL kanamycin at 37 °C while being shaken at 200 rpm until the OD_600_ reached 0.8, and protein expression was induced with 1 mM IPTG at 20 °C overnight with shaking at 200 rpm. Cells were collected by centrifugation, and the pellet was resuspended in 20 mM Tris-HCl and 8 M urea (pH 8). Following sonication (15 s on, 45 s off pulses with a 20% amplitude), centrifugation was used to remove cellular debris. The filtered supernatant was loaded onto two HisTrap 5 mL columns (Cytiva) in tandem that had been pre-equilibrated with binding buffer [20 mM Tris-HCl and 8 M urea (pH 8) supplemented with 15 mM imidazole]. Following a washing step with 10 column volumes of binding buffer, the fusion protein was eluted from the column with 5 column volumes of elution buffer [20 mM Tris-HCl and 8 M urea (pH 8) supplemented with 300 mM imidazole]. The eluted fusion protein was dialyzed overnight against 20 mM Tris-HCl (pH 8) to remove imidazole. TEV protease was then added to the fusion protein in a 1:15 molar ratio (TEV protease added to cleave the spider silk domain) at 4 °C overnight. Then, 7 M guanidine-HCl was added to the sample, and the mixture incubated on ice for at least 2 h before SEC using a Superdex 75 Increase pg 10/600 column (Cytiva) in 20 mM phosphate buffer supplemented with 200 μM EDTA (pH 8). The monomer concentration (in micromolar) was determined from the size exclusion chromatogram using the calculation
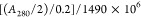
1where *A*_280_ is the absorbance at 280 nm of the elution peak of Aβ42, 0.2 is the path length (centimeters) of the ATKA Pure (Cytiva), and 1490 M^–1^ cm^–1^ is the molecular coefficient of Aβ42.

### Gel Electrophoresis and Western Blot Analysis

SDS–PAGE was performed using NuPAGE 4–12% Bis-Tris protein gels (Thermo Fisher Scientific) according to the manufacturer’s instructions. The PageRuler Plus prestained protein ladder (Thermo Fisher Scientific) was used to determine the molecular weights of the gel bands.

For Native PAGE, protein samples were prepared in native sample buffer [50 mM Tris-HCl, 5% glycerol, and 0.00125% bromophenol blue (pH 8.6)] and analyzed on Novex WedgeWell 4–20% Tris-Glycine protein gel (Thermo Fisher Scientific) using a native running buffer [2.5 mM Tris and 19.2 mM glycine (pH 8.3)]. For Western blotting, proteins were transferred to nitrocellulose (NC) membranes (Thermo Fisher Scientific) for 7 min at 20 V on an iblot2 gel transfer device (Thermo Fisher Scientific). Membranes were blocked with 5% (w/v) nonfat dried milk in Tris-buffered saline [20 mM Tris and 0.15 mM NaCl (pH 7.4)] containing 0.1% (v/v) Tween 20 (TBST) for 1 h at RT. After being washed three times with TBST, the membrane was incubated with rabbit monoclonal anti-α-syn antibody MJFR1 (Abcam) at a 1:1000 dilution in TBST overnight. After being washed three times with TBST, the membrane was incubated with a goat anti-rabbit secondary antibody Alexa Fluor 555 conjugated (Thermo Fisher Scientific) at a 1:2000 dilution in TBST for 1 h at RT. Three TBST washes followed, and the proteins were visualized by fluorescence scanning on a Typhoon Variable Mode Imager 9500 (GE Healthcare) using Cy3 (λ_ex_ = 532 nm; λ_em_ = 610 nm) filters to detect the Alexa Fluor 555 fluorophore.

### CD Spectroscopy

The far-ultraviolet CD spectra of 20 μM αSP1 in PBS or 15 μM αSP1 in 20 mM sodium acetate buffer and 150 mM NaCl (pH 4.8) were recorded at 37 °C using a Chirascan V100 circular dichroism spectrometer (Applied Photophysics). Five accumulations (PBS), or a single accumulation per hour for 15 h (pH 4.8), were performed in a 0.1 cm cuvette using a 0.5 nm step, 1 s per point, and a spectral range of 200–250 nm. A buffer spectrum was subtracted from each time point.

### ThT α-Syn and Aβ42 Fibrillation Assays Using Periplasmic Extracts or Pure Protein at Neutral pH

For the α-syn aggregation screening using periplasmic extracts, 30 μM WT α-syn was incubated in PBS in the presence of ∼75% (v/v) periplasmic extract, 12 μM ThT, and 0.02% (w/v) NaN_3_. A 150 μL portion of each sample (one replicate only) was loaded into a 96-well full-area plate (nonbinding, clear bottomed) and incubated at 37 °C for ∼40 h in a CLARIOstar Plus microplate reader (BMG Labtech).

Upon selection, purified αSP1 was incubated in the presence of 20 μM WT α-syn in PBS, 12 μM ThT, and 0.02% (w/v) NaN_3_ at various αSP1:α-syn molar ratios (0:1, 1:10, 1:20, 1:50, and 1:100). Then, 170 μL of each sample (three replicates) was loaded into a 96-well full-area plate (nonbinding, clear bottomed) and incubated at 37 °C for ∼140 h in a CLARIOstar Plus microplate reader.

Aggregation in both cases was stimulated through linear shaking (300 rpm, 300 s before each cycle) with the addition of a single glass bead (3 mm diameter) to each well. The fluorescence intensity was measured using spiral averaging (5 mm diameter) every 606 s using excitation 440 nm, dichroic 460 nm, and emission 480 nm filters, three gains, and 50 flashes per well.

For Aβ42, 1 μM protein monomer solutions in PBS were incubated in the presence of 20 μM ThT. Then, 180 μL of each sample (three replicates) was loaded into a 96-well full-area plate (nonbinding, clear bottomed) and incubated at 37 °C under quiescent conditions for 22.5 h in a CLARIOstar Plus microplate reader. The fluorescence intensity was measured using spiral averaging (3 mm diameter) using excitation 440 nm, dichroic 460 nm, and emission 480 nm filters, four gains, and 50 flashes per well.

To assess the efficacy of αSP1, 20 μM WT α-syn was aggregated into amyloid in the presence of increasing amounts of cytoplasmic extracts of *E. coli* BL21(DE3) cells with a range of αSP1 concentrations. Briefly, cells were grown at 37 °C until an OD_600_ of 0.7 while being shaken at 200 rpm overnight in LB with no antibiotics. Cells were collected by centrifugation, resuspended in buffer A [20 mM Tris-HCl and 1 mM EDTA (pH 8.0)] with a protease inhibitor tablet, and sonicated for 5 min with 15 s pulses and a 45 s rest. Cellular debris was cleared by centrifugation, and the supernatant was boiled at 80 °C for 20 min. This was then centrifuged at maximum speed (18 000 rpm) using a Sorvall Lynx 4000 centrifuge (Thermo Fisher Scientific) for 20 min, and the supernatant was collected. Streptomycin was gradually added to reach a final concentration of 10 mg/mL to precipitate DNA, and the mixture incubated at 4 °C for 20 min. After a final 30 min centrifugation step at 18 000 rpm, the supernatant was used as the cytoplasmic extract.

### Negative Staining and Transmission Electron Microscopy

Samples were spotted for 1 min on Formvar/Carbon-coated 300 mesh copper grids, after which excess sample was removed by blotting the grids dry with Whatman filter paper. Grids were then washed with water and stained with 2% (w/v) uranyl acetate. Grids were imaged on a T12 Spirit electron microscope (Thermo Fisher Scientific-FEI).

### ELISA for Assessing αSP1 Specificity and Binding to α-Syn Aggregates

To assess αSP1 specificity, 30 μM WT or ΔP1 α-syn monomers were immobilized onto a 96-well MaxiSorp ELISA plate (Nunc) and incubated at 4 °C. The plate was then washed three times with TBS [20 mM Tris (pH 7.4) and 100 mM NaCl] and blocked with 5% (w/v) BSA in TBS overnight. The plate was then washed six times with TBS and incubated with 30 μL of 1–10 μM αSP1 per well at room temperature for 1 h while being constantly shaken. The plate was then washed six times with TBS and incubated with 30 μL solutions of rabbit polyclonal six-His tag horseradish peroxidase (HRP) conjugated (Abcam) at a 1:4000 dilution in 5% (v/v) BSA-TBS for 1 h at room temperature while being constantly shaken. The plate was finally washed three times with TBS, twice with TBS supplemented with 0.02% (v/v) Tween 20, and three times again with TBS. The amount of bound αSP1 was quantified using the 1-Step Ultra TMB-ELISA substrate solution (Thermo Fisher Scientific) as per the manufacturer’s instructions, and the absorbance at 450 nm was read using the CLARIOstar plate reader (BMG Labtech). To assess the binding of αSP1 to α-syn aggregates, 20 μM WT α-syn was aggregated as described above; then, 20 μL aliquots were taken at different times (0, 5, 10, 24, and 48 h) and immobilized on a 96-well Maxisorp ELISA plate, and subsequent steps were carried out as described above with 2 μM αSP1.

To assess the binding of αSP1 to α-syn monomers, stabilized oligomers, and fibrils, 80 pmol of αSP1 was immobilized on the plate and 160 pmol of each α-syn aggregate was added after the blocking step described above. The level of binding was assessed by the anti-α-syn-HRP (Biolegend) antibody reacting with the Ultra TMB-ELISA substrate solution as described above. For this assay, blocking and antibody dilutions were carried out using 5% (v/v) goat serum in TBS.

### Secondary Nucleation Assay

αSP1 was incubated in the presence of 20 μM monomeric WT α-syn, 20 nM WT α-syn PFFs, 20 μM ThT, and 0.02% (w/v) NaN_3_ at various molar ratios of αSP1 to monomeric α-syn (0:1, 1:20, 1:50, 1:100, 1:250, and 1:500). Controls in the absence of PFFs (all ratios) and in the absence of monomeric α-syn (highest ratio) alongside fibril seeds alone and αSP1 alone (highest ratio) were also included. The reaction was carried out in 20 mM sodium acetate [150 mM NaCl (pH 4.8)]. All protein stocks were dialyzed or diluted in 20 mM sodium acetate [150 mM NaCl (pH 4.8)] immediately before use. Then, 170 μL of each sample (three replicates) was loaded into a 96-well full-area plate (nonbinding, clear bottomed) and incubated at 37 °C quiescently for ∼100 h in a FLUOstar Omega microplate reader (BMG Labtech). The fluorescence intensity was measured using spiral averaging (3 mm diameter) every 420 s using excitation 440 nm, dichroic 460 nm, and emission 480 nm filters, four gains, and 50 flashes per well. All data are background corrected.

### Förster Resonance Energy Transfer Binding Assay

To obtain the *K*_d_ for binding of αSP1 to α-syn monomers, oligomers, and fibrils at pH 7.4, FRET binding assays were performed, in which α-syn and αSP1 were labeled with Alexa Fluor 488 and Atto 647 N, respectively.

Labeled α-syn monomers were obtained by conjugation of A140C α-syn with Alexa Flour 488 C5-maleimide (Thermo Fisher Scientific). A140C α-syn was buffer-exchanged using Zeba Spin Desalting Columns (7K molecular weight cutoff) (Thermo Fisher Scientific) to remove the DTT from the storage solution. The reaction was carried out according to the manufacturer’s instructions.

Labeled α-syn oligomers were obtained by lyophilizing a 1:10 solution of labeled and unlabeled α-syn monomers overnight. The lyophilized protein was then resuspended in the same volume of PBS as before lypholization to maintain the 1:10 labeled:unlabeled molar ratio (80 μM:800 μM) and incubated at 37 °C for 20–24 h. Ultracentrifugation was carried out as described in Generation of α-Syn-Stabilized Oligomers. Then, monomers were removed by four centrifugations with a 100 kDa molecular cutoff filter.

Labeled fibrils were obtained by seeding 100 μM 488 monomers with 10 μM PFFs in PBS under quiescent conditions for 48 h. Labeled fibrils were collected by centrifugation at maximum speed (16900*g*) for 30 min and washed with PBS to remove any soluble species. For labeled oligomers and fibrils, the concentration was determined as described in Generation of α-Syn-Stabilized Oligomers and Generation of α-Syn Preformed Fibrils, accounting for the fluorophore absorbance and a correction factor, as per the manufacturer’s instructions. αSP1 was labeled with an NTA Atto 647 N kit (Sigma-Aldrich) as per the manufacturer’s instructions. For experiments at pH 4.8, α-syn monomers and αSP1 were buffer exchanged into 20 mM acetic acid buffer (11.7 mM sodium acetate and 8.3 mM acetic acid) supplemented with 150 mM sodium chloride before labeling. As the NTA Atto 647 N may not be compatible with pH 4.8, αSP1 was labeled targeting the native Cys residues with an Alexa Fluor 633 C5-maleimide kit (Thermo Fisher Scientific), as per the manufacturer’s instructions. As a comparison, a titration experiment at pH 7.4 was performed using αSP1 labeled with the same kit. A range of labeled α-syn monomer, oligomer, and fibril concentrations were added to labeled 0.5 μM αSP1 in triplicate, and the fluorescence intensity was measured using excitation and emission wavelengths of 493 and 672 nm, respectively. Measurements were taken by using a ClarioStar Plus microplate reader (BMG Labtech). Fluorescence data were plotted and analyzed by using GraphPad Prism version 9.3.1 (GraphPad Software). Fluorescence data were fitted using the following one-site specific binding model.
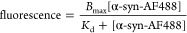
2where *B*_max_ is the predicted maximum fluorescence. The *K*_d_ was constrained to be shared across independent replicates. The fluorescence data were then converted into a fraction of bound ligand, i.e., fraction bound, by diving them for the fitted *B*_max_. This was then fitted by using the aforementioned binding model in which *K*_d_ was shared across the independent replicates, and the plateau was set to be 1.

### MTS Cell Viability Assay

SH-SY5Y cells were cultured in RPMI medium supplemented with 10% (v/v) fetal bovine serum under the 5% CO_2_ condition as previously described.^58^ Cells were not used past passage 19. For MTS assays, cells were plated at a density of 10 000 cells per well in 96-well plates 24 h prior to being incubated with protein samples. Cells were incubated with a final concentration of 10 μM α-syn aggregated as described above in the absence or presence of αSP1 at a 1:20 sybody:α-syn ratio. Protein samples were diluted in a serum-free medium. After incubation for 24 h, the culture medium was replaced with fresh serum-free medium and the MTS assay (Promega) was carried out as per the manufacturer’s instructions. Absorbance readings were taken at 490 nm by using a ClarioStar Plus microplate reader (BMG Labtech). Absorbance values from controls with only medium were subtracted from each condition, and data were normalized to cells treated with α-syn aggregated in the absence of αSP1. Statistical analysis was carried out by one-way ANOVA with Tukey’s multiple-comparison test.

### Caspase-Glo 3/7 Assay

SH-SY5Y cells were cultured, plated, and incubated with samples from 72 h aggregation assays, as described above (see MTS Cell Viability Assay). After incubation for 24 h, caspase 3/7 activation was measured as an indicator of apoptosis using the Caspase-Glo 3/7 Assay System (Promega), as per the manufacturer’s instructions. Luminescence readings were taken using a ClarioStar Plus microplate reader (BMG Labtech). Luminescence values from controls with only medium were subtracted from each condition, and data are expressed normalized to cells incubated with α-syn aggregated in the absence of αSP1. Statistical analysis was carried out by one-way ANOVA with Tukey’s multiple-comparison test.

## Supporting Information Available

Screening of the ability of sybody candidates to inhibit the amyloid aggregation of α-syn (Figure S1), sequences of sybody αSP1 (Figure S2), purification of αSP1 (Figure S3), far-ultraviolet CD spectrum of αSP1 (Figure S4), ELISA to verify the binding of αSP1 to the P1 region (Figure S5), α-syn soluble fraction analysis at the end of an aggregation (Figure S6), caspase 3/7 activation in SH-SHY5Y cells exposed to α-syn aggregates formed in the absence or presence of αSP1 (Figure S7), DLS of α-syn preformed fibrils at pH 4.8 (Figure S8), time course of the far-ultraviolet CD spectra of αSP1 at pH 4.8 and 37 °C (Figure S9), Native PAGE and Western blotting of unlabeled α-syn monomers and aggregated species used for the ELISA (Figure S10), ELISA to measure the binding of αSP1 to monomeric, oligomeric, and fibrillar α-syn (Figure S11), and FRET-based titrations of αSP1-AF633 to monomeric α-syn-AF488 at pH 7.4 and 4.8 (Figure S12) (PDF)

## Abbreviations Used

Aβ: amyloid-β peptide
Aβ42: 42-residue variant of Aβ
BBB: blood–brain barrier
BSA: bovine serum albumin
CD: circular dichroism
CDR: complementarity-determining region
DLS: dynamic light scattering
DTT: dithiothreitol
EDTA: ethylenediaminetetraacetic acid
ELISA: enzyme-linked immunosorbent assay
Fc: fragment crystallizable (domain)
FDA: Food and Drug Administration
FRET: Förster resonance energy transfer
FWRs: framework regions
FX: fragment exchange (cloning)
HRP: horseradish peroxidase
IMAC: immobilized metal affinity chromatography
LB: lysogeny broth
MSA: multiple-system atrophy
MTS: 3-(45-dimethylthiazol-2-yl)-5-(3-carboxymethoxyphenyl)-2-(4-sulfophenyl)-2*H*-tetrazolium
NAC: non-amyloid-β component
NC: nitrocellulose
OD: optical density
PAGE: polyacrylamide gel electrophoresis
PBS: phosphate-buffered saline
PCR: polymerase chain reaction
PD: Parkinson’s disease
PFFs: preformed fibrils
RT-PCR: reverse transcription polymerase chain reaction
SD: standard deviation of the mean
sdAb: single-domain antibodies
SDS: sodium dodecyl sulfate
SEC: size exclusion chromatography
SEM: standard error of the mean
TBS: Tris-buffered saline
ThT: thioflavin T
WT α-syn: wild-type α-synuclein
α-syn: α-synuclein
