# Supplementary material for: A Potent Sybody Selectively Inhibits α-Synuclein Amyloid Formation by Binding to the P1 Region
Source: J Med Chem. 2024 Jun 6;67(12):9857–68. doi: 10.1021/acs.jmedchem.3c02408 (PMC11215725; doi:10.1021/acs.jmedchem.3c02408)
Supplement: Supplementary file 1 — jm3c02408_si_001.pdf [file jm3c02408_si_001.pdf]

A Potent Sybody Selectively Inhibits α-Synuclein Amyloid Formation by Binding to the P1 Region

Dimitra Gialama,[1,§] Devkee M. Vadukul,[1] Rebecca J. Thrush,[1,2] Sheena E. Radford,[3] and Francesco A. Aprile[1,2*]

[1]Department of Chemistry, Molecular Sciences Research Hub, Imperial College London, London W12 0BZ, UK
[2]Institute of Chemical Biology, Molecular Sciences Research Hub, Imperial College London, London W12 0BZ, UK

[3]Astbury Centre for Structural Molecular Biology, School of Molecular and Cellular Biology, University of Leeds, Leeds LS2 9JT

[§]present address: Biomedical Sciences Research Center "Alexander Fleming", Vari 16672, Greece

*Author to whom correspondence should be addressed.
Email: f.aprile@imperial.ac.uk, Phone: +44 (0)20 7594 5545

Table of Contents

**Contents**	**Page**
**Figure S1.** Screening of the ability of sybody candidates to inhibit the amyloid aggregation of α-syn.	S3
**Figure S2.** Sequences of the sybody αSP1.	S4
**Figure S3.** Purification of αSP1.	S5
**Figure S4.** Far-UV CD spectrum of αSP1.	S6
**Figure S5.** ELISA assay to verify αSP1 binding to the P1 region.	S7
**Figure S6.** α-Syn soluble fraction analysis at the endpoint of an aggregation.	S8
**Figure S7.** Caspase 3/7 activation in SH-SHY5Y cells exposed to α-syn aggregates formed in the absence or presence of αSP1.	S9
**Figure S8.** DLS of α-syn preformed fibrils at pH 4.8.	S10
**Figure S9.** Time-course of the far-UV CD spectra of αSP1 at pH 4.8 and 37 °C	S11
**Figure S10.** Native Page and western blotting of unlabelled α-syn monomers and aggregated species used for ELISA.	S12
**Figure S11.** ELISA to measure the binding of αSP1 to monomeric, oligomeric, and fibrillar α-syn.	S13
**Figure S12.** FRET-based titrations of αSP1-AF633 to monomeric α-syn-AF488 at pH 7.4 and 4.8.	S14

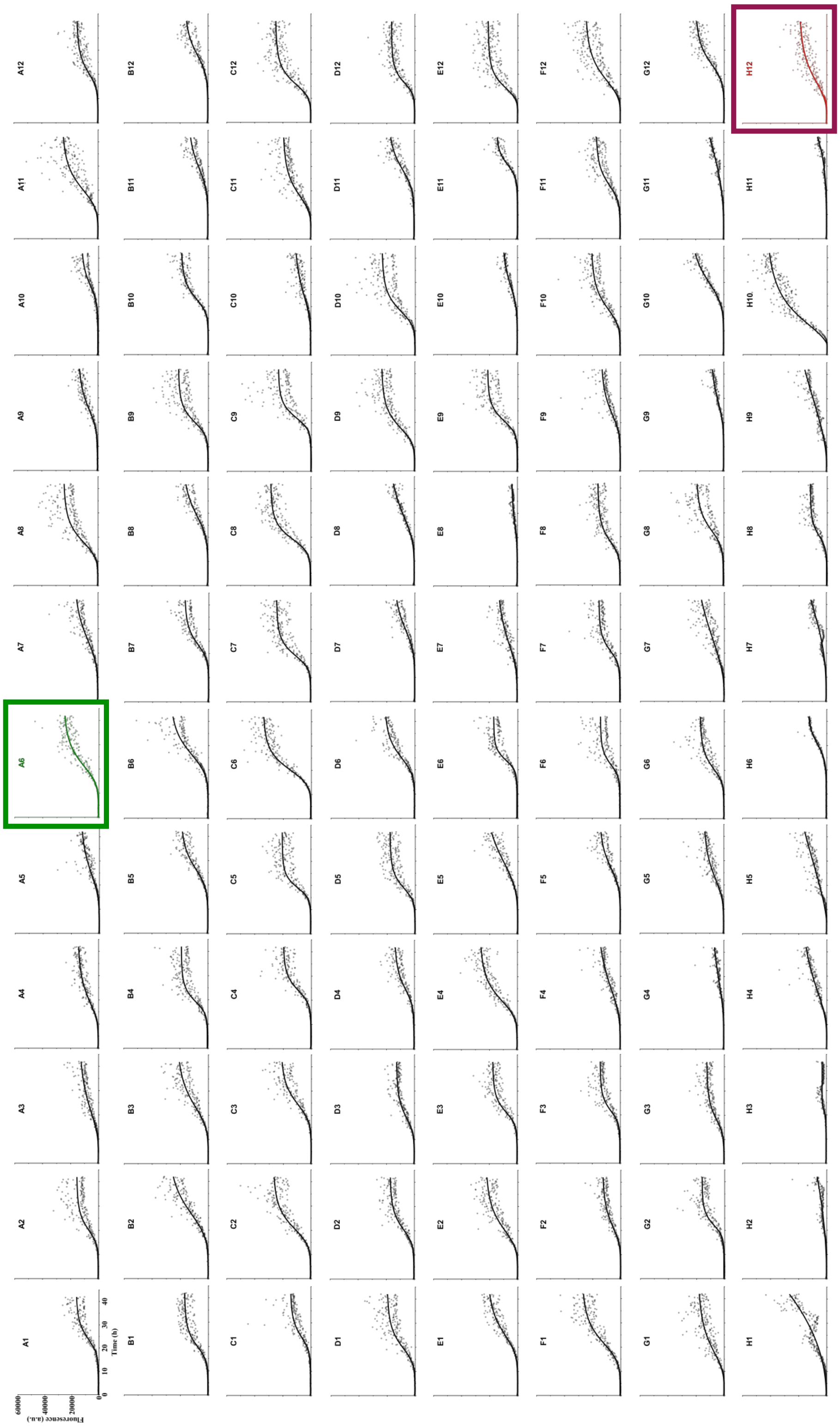

Figure S1. Screening of the ability of sybody candidates to inhibit the amyloid aggregation of α-syn. Raw data of the screening shown in Figure 2. αSP1 (clone A6) is shown in green and the control experiment with a non-expressing crude extract is shown in red (CTRL in Figure 2).

Protein sequence αSP1

MSKYLLPTAAAGLLLLAAQPAMAGSSSQVQLVESGGGLVQAGGSLRLSCAASGFPV**RHQWME**WYRQ
APGKEREWVAAI**SSFGQWTK**YADSVKGRFTISRDNAKNTVYLQMNSLKPEDTAVYYC**VVEVGYEYY**GQ
GTQVTVSAGRAG**EQKLISEEDL**NSAVD**HHHHHH**

DNA sequence αSP1

ATGAGTAAATATCTGCTGCCGACCGCAGCAGCGGGTCTGCTGCTGCTGGCAGCCCAGCCTGCAATG
GCCGGCTCTTCAAGTCAGGTTCAGCTGGTTGAGAGCGGTGGTGGCCTGGTCCAAGCTGGCGGTTCG
CTGCGTCTGAGCTGCGCCGCAAGCGGTTTCCCGGTG**AGGCATCAGTGGATGGAA**TGGTATCGTCAG
GCCCCGGGCAAAGAACGTGAGTGGGTCGCGGCGATT**TCTAGCTTCGGTCAATGGACGAAA**TACGC
AGATTCTGTTAAGGGCCGCTTTACCATCAGCCGCGACAACGCGAAGAATACGGTCTATTTGCAGATG
AATAGCCTGAAACCGGAAGATACCGCGGTTTACTACTGT**GTTGTGGAAGTGGGTTACGAATACTAC**
GGCCAAGGTACCCAAGTGACTGTGAGCGCAGGAAGAGCTGGC**GAACAAAAACTCATCTCAGAAGA**
GGATCTGAATAGCGCCGTCGAC**CATCATCATCATCATCAT** **TGA (stop codon)**

PelB cleaved upon sorting to the periplasmic space
CDR1 complementarity determining region 1*
CDR2 complementarity determining region 2*
CDR3 complementarity determining region 3*
Myc tag tag for detection
His tag tag for purification
*The varied regions are colored in yellow, cyan, and purple.

Figure S2. Sequences of the sybody αSP1. The different sequence elements are indicated with different colors.

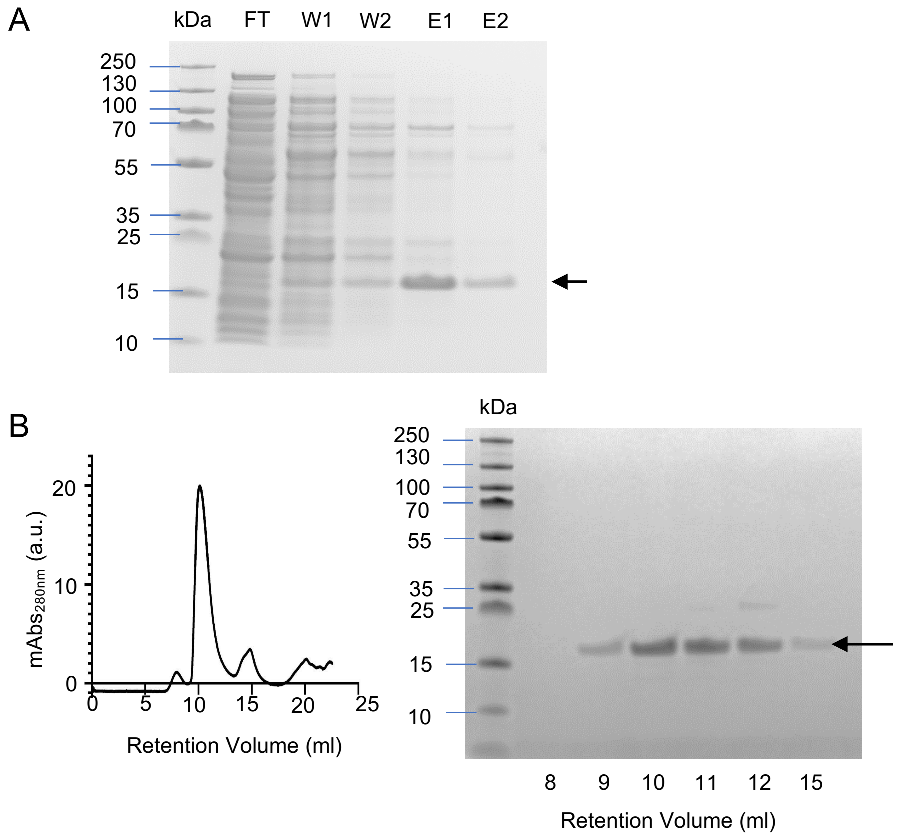

Figure S3. **Purification of αSP1**. **A)** SDS-PAGE of the IMAC purification. FT: flow through, W1: wash 1, W2: wash 2, E1: elution 1, E2: elution 2. The arrow depicts the mobility of αSP1. **B)** Representative chromatogram (left) and SDS-PAGE (right) of the SEC. In the SDS-PAGE, the retention volume at which collection started is reported for each fraction. The fraction volume was 1 ml. αSP1 is marked with an arrow.

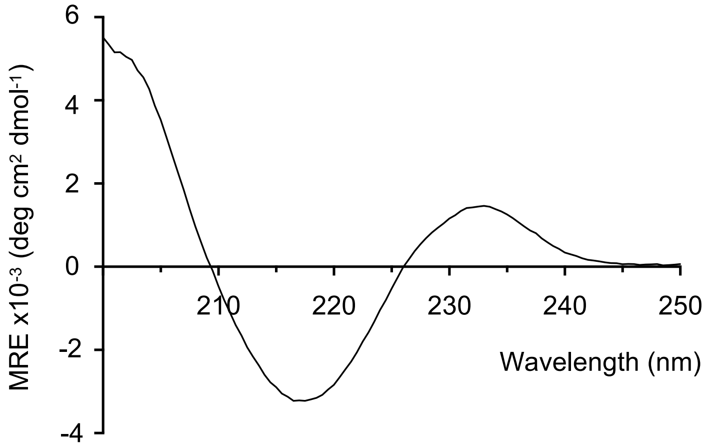

Figure S4. Far-UV CD spectrum of αSP1. The spectrum was collected at 20 μM of αSP1 concentration in PBS pH 7.4 at 37 °C.

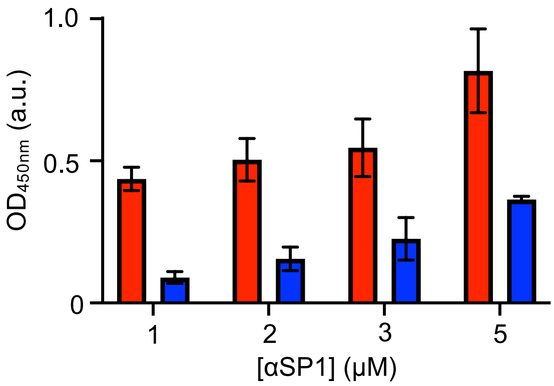

Figure S5. ELISA assay to verify αSP1 binding to the P1 region. Increasing concentrations of αSP1 were used with WT α-syn monomer (red) and an α-syn variant lacking the P1 region (blue), α-syn ΔP1. The error bars represent the SD of 3 technical replicates.

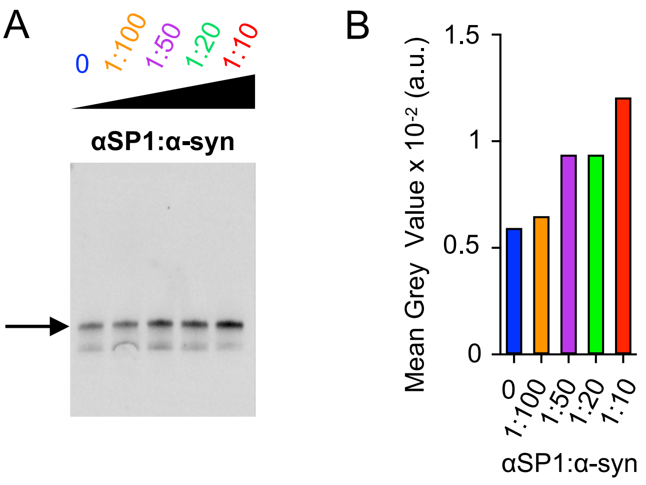

Figure S6. α-Syn soluble fraction analysis at the endpoint of an aggregation. A) SDS-PAGE and Western blot and **B)** quantification of the bands using ImageJ.

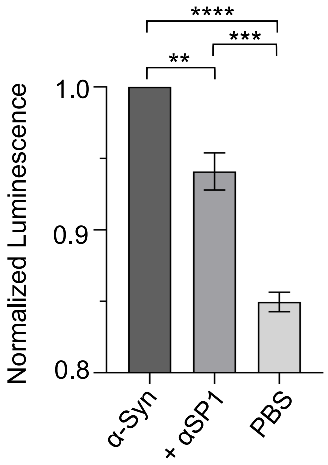

Figure S7. Caspase 3/7 activation in SH-SHY5Y cells exposed to α-syn aggregates formed in the absence or presence of αSP1. Cells were treated with α-syn aggregated in the absence or presence of αSP1 (molar ratio of 1:20, [αSP1]: [α-syn]) for 72 h. Luminescence values of the different conditions were normalized over those of the cells incubated with only α-syn. The averages of 3 biological replicates are shown. The error bars represent SEM of the biological replicates. Each biological replicate is the average of 4–10 technical replicates. Statistical analysis was carried out by one-way ANOVA with Tukey's multiple comparison test where $0.01 \geq P > 0.001$ (**), $0.001 \geq P > 0.0001$ (***), and $P \leq 0.0001$ (****).

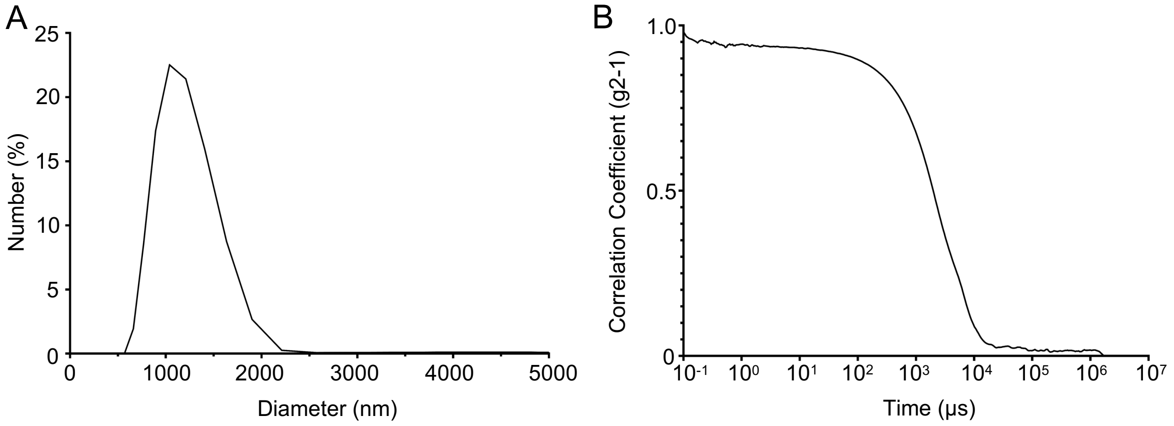

Figure S8. DLS of α-syn preformed fibrils at pH 4.8. A) Representative number distribution and **B)** correlogram are shown.

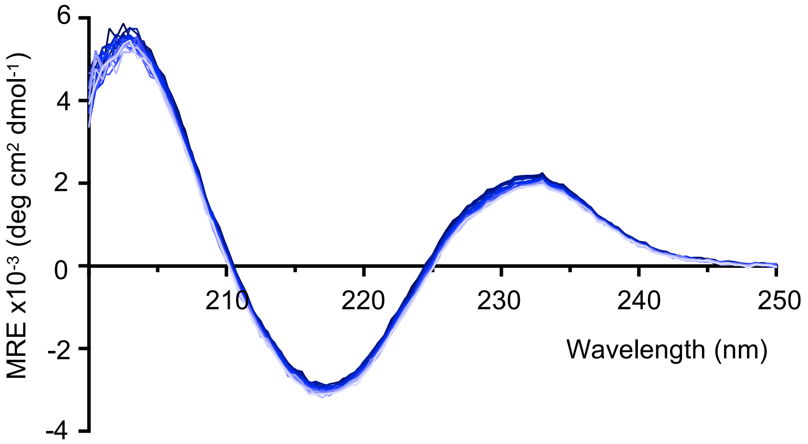

Figure S9. Time-course of the far-UV CD spectra of αSP1 at pH 4.8 and 37 °C. Each spectrum was taken every hour from 0–19 h (dark to light blue).

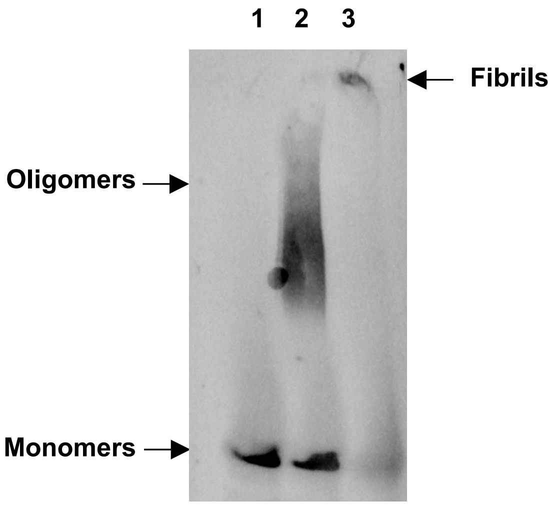

Figure S10. Native Page and western blotting of unlabelled α-syn monomers and aggregated species used for ELISA. Lane 1, monomeric sample; lane 2, sample enriched in oligomers; lane 3, sample enriched in amyloid fibrils.

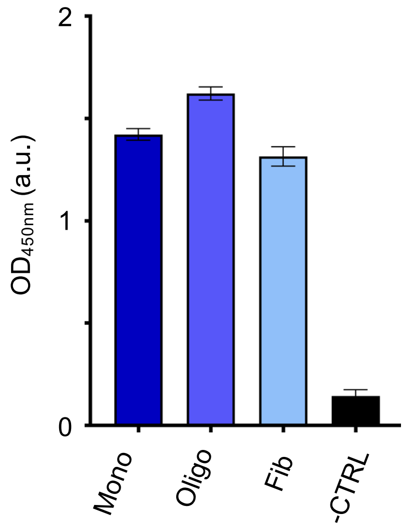

Figure S11. ELISA to measure the binding of αSP1 to monomeric (mono), oligomeric (oligo), and fibrillar (fib) α-syn. The negative control was performed by blocking the wells with BSA in the absence of α-syn to show the level of non-specific binding. Error bars are the SD of 3 technical replicates.

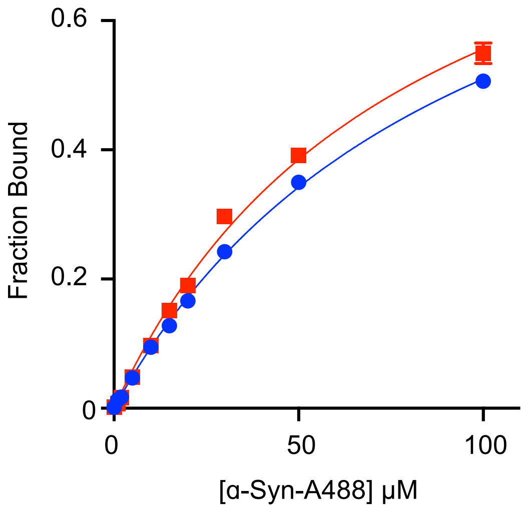

Figure S12. FRET-based titrations of αSP1-AF633 to monomeric α-syn-AF488 at pH 7.4 (in blue) and 4.8 (in red). Similar K_d (96±0.7 μM at pH 7.4 and 80±1.2 μM at pH 4.8) were obtained. Error bars are the SD of 3 technical replicates.
